# Tracking of health-related physical fitness in adolescent girls: a 3-year follow-up study

**DOI:** 10.1186/s12887-022-03305-2

**Published:** 2022-04-29

**Authors:** Mario Kasović, Ana Oreški, Tomaš Vespalec, Kateřina Jenčíková, Lovro Štefan

**Affiliations:** 1 Department of General and Applied Kinesiology, Faculty of Kinesiology, University of Zagreb, 10 000 Zagreb, Croatia; 2 Department of Sport Motorics and Methodology in Kinanthropology, Faculty of Sports Studies, Masaryk University, 62 500 Brno, Czech Republic; 3 Secondary School ‘Gospodarska škola Varaždin’, 42 000 Varaždin, Croatia; 4 Department of Research and Examination (RECETOX), Faculty of Science, Masaryk University, 62 500 Brno, Czech Republic

**Keywords:** Performance, Stability, Youth, Secondary school, Generalized estimating equations

## Abstract

**Background:**

Evidence has shown that higher levels of physical fitness (PF) in youth have beneficial effects on adult health-related outcomes. However, the tracking of separate PF components during adolescence has been less studied. Since PF often starts to rapidly decline during adolescence, it is necessary to provide information regarding critical time-point for interventions. This study aimed to analyze the extent of tracking the components of PF through PF tests.

**Methods:**

In this longitudinal study, we recruited 240 adolescent girls with recoded data at 2 time-points (15 y and 17 y). PF included body composition (fat mass), explosive power of lower extremities (standing broad jump), muscle endurance of the trunk (sit-ups in 60 s), flexibility (sit-and-reach test), muscle endurance of lower extremities (squats in 60 s), aerobic endurance (the 800 m run test) and speed endurance (the 400 m run test). Tracking coefficients were calculated using generalized estimating equations. Tertiles (high, moderate and low) were calculated for each fitness component.

**Results:**

The highest tracking coefficients between the two time-points were found for explosive power of lower extremities (*β* = 0.98), followed by flexibility (*β* = 0.89), body composition (*β* = 0.88), speed endurance (*β* = 0.86), aerobic endurance (*β* = 0.75), muscle endurance of lower extremities (*β* = 0.65), and muscle endurance of the trunk (*β* = 0.51). Tertile ratings remained stable across the two time-points.

**Conclusions:**

Moderate to high tracking of PF in adolescent girls suggests that interventions aiming to increase the level of PF should probably begin in early adolescence.

## Background

Physical fitness (PF) is a multi-dimensional, well-documented marker of health [1]. It is often described as ‘the capacity to perform physical activity and refers to a full range of physiological and psychological qualities’ [2]. Main components of PF include: (i) cardiorespiratory fitness, (ii) muscular fitness, (iii) speed and (iv) body composition [1]. In youth, maintenance of lower levels of PF may have both short- and long-term consequences on health, including higher incidence of cardiorespiratory and metabolic risk factors during young age and in adulthood, which leads to premature all-cause mortality [1, 3, 4].

Evidence suggests that children and adolescents between ages 6 and 17 should participate in muscle-strengthening activities at least 2 or more days per week at moderate or greater intensity and in at least 60 min of mostly aerobic moderate-to-vigorous intensity activities on a daily basis [5]. Despite health-related benefits and efforts to promote PF [1], studies have shown that the level of PF in youth has consistently declined over the past three decades in the United States [6–8] and Europe [9, 10]. Thus, by screening and monitoring PF across the lifespan, professionals would be able to implement special interventions and policies in everyday settings.

Given the importance of lifelong PF for health, it is assumed that such construct tracks well from point-to-point. Tracking can be defined in two ways: (i) ‘a tendency of individuals to maintain their rank within a certain group over a period of time’ [11] and (ii) 'the ability to predict future observations based on earlier values’ [12].

Although collecting longitudinal data has some limitations in terms of high costs, time and drop-out rates [13], several longitudinal PF analyses of different components are available in the literature [13–25]. In general, moderate to high tracking of PF has been observed, with the results being strongly dependent on sex [14–16], the follow-up period [17, 18], the number of time-points being measured [19–23] and the selection of different fitness tests [18, 19, 24].

In Croatia, the most recent study has shown negative secular trends of health-related PF; from 1999 to 2014 body mass index increased, while cardiorespiratory and muscular fitness decreased in both sexes [26]. A common perception has been embraced that more time spent in sedentary behavior, lack of physical activity and consuming fat-rich food are the most prevalent factors for lower PF levels [27]. Since the level of PF starts to rapidly decline during adolescence, it is valuable to examine how different fitness components track during this critical period.

Therefore, the main purpose of the study was to analyze the extent of tracking of several PF tests. We hypothesized that the performance on PF measures would track moderate to high across age comparisons and the maintenance of fitness tertile ratings would remain stable over time.

## Methods

### Study participants

In this longitudinal study, participants were adolescent girls measured at two time-points (15 y and 17 y). Specifically, out of ten secondary schools in the 'Varaždin' county (≈45.000 inhabitants), 3 schools were randomly selected. Randomization of schools was done with replacement by drawing school codes on slips of paper from a box, with each school having equal probability of selection. At the second stage, four classes within each school were selected, which gave a total of twelve classes and 286 girls. Of them, nineteen did not attend physical education classes when the measurements were conducted and thirty-seven had a measurement at only one time-point. Our final sample was consisted of 240 adolescent girls who were measured in all PF components at two time-points. To confirm the representativeness of the sample, using a statistical power of 85%, margin of error of 5%, and the population of secondary school students in the ‘Varaždin’ county of ≈7.000, the required sample size was estimated at 202 adolescent girls. Parent of each participant and all participants gave informed written consent before enrollment into the study for participation. Analyses and procedures performed in the study were conducted in accordance with the Declaration of Helsinki [28] and approved by the Ethics Committee of the Faculty of Kinesiology, University of Zagreb, Croatia.

### Health -related physical fitness

To assess the level of health-related PF, the following tests were applied: (i) fat mass (body composition), (ii) standing broad jump (explosive power of lower extremities), (iii) sit-ups in 60 s (muscle endurance of the trunk), (iv) sit-and-reach test (flexibility), (v) squats in 60 s (muscle endurance of lower extremities), (vi) the 800 m run test (aerobic endurance), and (vii) the 400 m run test (speed endurance). The methodology of data collection of each test has been described previously [29–31]. Prior the study, physical education teachers from each school were instructed, how to conduct the tests. All tests were assessed between September and October at both time-points and were performed during physical education classes. To avoid fatigue, the protocol was split into two non-consecutive days during the morning hours between 8:00 am and 11:00 am. On the first day of measurement, the tests were performed in following order: 1) fat mass, 2) standing broad jump, 3) sit-ups in 60 s, 4) sit-and-reach test and 5) squats in 60 s. Before every test, each participant had ≈15 min of resting period. On the second day, the 800 m run, and the 400 m run tests were applied. Fat mass was measured using bioelectrical impedance analysis for three consecutive times (Omron BF500Body Composition Monitor, Omron Medizintechnik, Vernon Hills, IL, USA). The reliability for three measurements was excellent (Cronbach’s alpha > 0.90). Standing broad jump tests jumping distance from a standing start (‘frog leap’), where each participant bends knees parallel to the ground and swings both arms, jumping vigorously as far as possible, trying to land with their feet together and stay upright [29]. Sit-up test evaluates muscle endurance of the trunk as number of sit-ups completed from lying position (knees bent at a 90°) in 60 s [29]. Sit-and reach test assesses the level of flexibility, by sitting on the floor or a mat, legs straight under the angle of 90°, the person being tested reached forward with the arms (hands overlapping). The distance of reach was measured in centimeters using a measuring non-elastic tape attached on the floor [30]. Squats in 60 s measures muscle endurance of lower extremities. The subject stood in a position where legs were spread in shoulder – width, hills were put at the edge of the mat and hands were relaxed in their natural position. During the performance, the subject went down till the position where the tips of both hands touched the ground and went up with both legs in full extension. The total amount of correctly performed squats in one minute was the score (reps) [31]. The 800 m run test assesses aerobic capacity. Participants were asked to complete the 800-m course in the quickest possible time around the standardized track and field running track 400 m in length. On the command ‘1,2,3 and go’, all participants began to run at their own pace. If a child had any kind of problem during the test, they were told to slow down or stop the test. Each trial was done with small groups of five to perform the test, to prevent from competition [32]. The final time in minutes was the score. Finally, the 400 m run test was used to assess the level of speed endurance. The same protocol as for the 800 m run test was used and the final score was recorded in minutes.

### Data analysis

Basic descriptive statistics are presented as mean and standard deviation (SD). The Kolmogorov–Smirnov tests showed that data were normally distributed. Baseline and follow-up differences were calculated using paired sample t-test. Cohen *d* effect sizes (ES) were also calculated to determine the magnitude of the group differences in health-related PF. ES was classified as follows: < 0.2 was defined as trivial; 0.2–0.5 was defined as moderate; 0.5–0.8 was defined as large; and > 0.8 was defined as very large [33]. Tracking of PF was assessed using generalized estimating equations. To describe the extent of tracking, we calculated a stability coefficient, the value of the outcome was regressed on the predictor [34]. The coefficient ranges from 0 to 1, with 1 indicating perfect tracking and 0 indicating no tracking. Participants’ scores in PF test at both time-points were classified into tertiles (high, moderate and low). Cross-tabulation matrices, percent agreements and kappa statistics were used to assess the ranking stability over time. Kappa tracking coefficients were classified as low (*r* < 0.3), moderate (*r* = 0.3–0.6), or moderately high (*r* > 0.6) [11]. Two-sided *p*-values were used, and significance was set at α < 0.05. All the analyses were calculated in Statistical Packages for Social Sciences v.23 (SPSS, Chicago, IL, United States).

## Results

Basic descriptive statistics are presented in Table 1. Although significant, small effect sizes were observed for height and weight. Over a 3-year period, fat mass significantly increased, while the level of explosive power of lower extremities (standing broad jump), muscle endurance of the trunk (sit-ups in 60 s), flexibility (sit-and-reach test), muscle endurance of lower extremities (squats in 60 s), aerobic capacity (the 800 m run test) and speed endurance (the 400 m test) decreased. Moderate-to-large effect sizes were presented for PF; the largest changes for aerobic capacity, speed endurance and flexibility were observed, followed by flexibility, muscle endurance of lower extremities and muscle endurance of the trunk. Body composition and explosive power of lower extremities exhibited the lowest effect sizes during the follow-up period of three years.

**Table 1:** Descriptive statistics of the study participants at baseline and follow-up (*N* = 240)

Study variables	Baseline	Follow-up	∆ (%)	ES	*p*-value
	Mean (SD)	Mean (SD)			
**Stature (cm)**	165.8 (7.3)	167.6 (7.5)	1.1	0.24	< 0.001
**Weight (kg)**	60.2 (14.0)	62.3 (14.8)	3.5	0.15	< 0.001
**Fat mass (%)**	27.0 (10.5)	29.8 (10.1)	10.4	0.27	< 0.001
**Standing broad jump (cm)**	178.5 (28.3)	170.9 (28.1)	-4.3	-0.27	< 0.001
**Sit-ups (reps in 60 s)**	55.4 (11.0)	51.2 (10.1)	-7.6	-0.40	< 0.001
**Sit-and-reach test (cm)**	69.4 (8.9)	64.5 (9.6)	-7.1	-0.53	< 0.001
**Squats (reps in 60 s)**	48.7 (8.0)	44.9 (7.9)	-7.8	-0.48	< 0.001
The 800 m run test (min)*	4.49 (0.78)	4.99 (0.78)	11.1	0.64	< 0.001
The 400 m run test (min)*	1.47 (0.31)	1.64 (0.38)	11.6	0.49	< 0.001

^*^Higher score denotes poorer performance, that is, more time is needed to complete the task in min

*p* < 0.05

Beta coefficients for the seven fitness tests appeared in Table 2. Tracking coefficients for the whole sample were significant at *p* < 0.001. All fitness components exhibited moderate-to- high tracking. The strongest tracking coefficients were obtained for explosive power of lower extremities, flexibility, body composition and speed endurance, while aerobic capacity and muscle endurance of lower extremities exhibited somewhat lower values. Finally, muscle endurance of the trunk showed moderate tracking characteristics over time.

**Table 2:** Tracking coefficients for each physical fitness component between the 2 time-points (*N* = 240)

Study variables	Tracking coefficient	95% CI	*p*-value
Fat mass (%)	0.88	0.84 to 0.92	< 0.001
Standing broad jump (cm)	0.98	0.97 to 0.99	< 0.001
Sit-ups (reps in 60 s)	0.51	0.41 to 0.61	< 0.001
Sit-and-reach test (cm)	0.89	0.86 to 0.92	< 0.001
Squats (reps in 60 s)	0.65	0.55 to 0.74	< 0.001
The 800 m run test (min)	0.75	0.67 to 0.84	< 0.001
The 400 m run test (min)	0.86	0.80 to 0.90	< 0.001

*p* < 0.05

The maintenance in a specific group (high, medium and low) is presented in Table 3. For body composition, 88.5% of 15-year-old girls with high fat mass remained in the same category at follow-up. Most notably, 23.8% of girls with low fat mass at baseline moved to ‘medium’ category. For explosive power of lower extremities, most of the participants categorized in ‘high’ and ‘medium’ categories remained in the same rank, while 22.2% of them being categorized in ‘low’ tertile increased their values to ‘medium’ category. The poorest persistence in muscle endurance of the trunk was observed; i.e. 28.6% of girls in ‘high’ tertile decreased their performance and move down to ‘medium’ rank. For the sit-and-reach test, 10.3% of girls in the ‘medium’ tertile at baseline decreased their flexibility performance and moved down to the lowest tertile. The largest changes between the tertiles were observed for muscle endurance of lower extremities, that is, 38.5% of girls moved from ‘high’ to ‘medium’ tertile rank. For aerobic capacity, 36.8% of girls in the highest tertile moved down to ‘medium’ tertile and 20.0% of those being categorized in ‘low’ tertile moved up in ‘medium’ tertile. Finally, 22.2% of girls in ‘high’ tertile and 18.2% of girls in ‘low’ tertile at baseline moved in ‘medium’ tertile. The strongest average agreement was indicated for explosive power of lower extremities, with slightly lower values being observed for flexibility, body composition and speed endurance. Similar percentage of agreement was shown for muscle endurance of lower extremities, aerobic endurance and muscle endurance of the trunk (< 70.0%). Kappa statistics showed moderate-to-high stability of tertile ratings (kappa = 0.43 to 0.86, *p* < 0.001).

**Table 3:** Girls’ maintenance of physical fitness tertile classification from baseline to follow-up (*N* = 240)

Baseline	Follow-up				
**Fat mass (%)**	High	Medium		Low	
High	88.5%	11.5%		0.0%	
Medium	5.2%	87.1%		7.8%	
Low	0.0%	23.8%		76.2%	
Kappa statistics (*p*-value)	0.75 (< 0.001)				
Agreement	83.9%				
**Standing broad jump (cm)**					
High	100.0%	0.0%		0.0%	
Medium	0.0%	92.9%		7.1%	
Low	0.0%	22.2%		77.8%	
Kappa statistics (*p*-value)	0.86 (< 0.001)				
Agreement	90.2%				
**Sit-ups (reps in 60 s)**					
High	61.9%	28.6%		9.5%	
Medium	19.0%	64.3%		16.7%	
Low	17.6%	17.6%		64.7%	
Kappa statistics (*p*-value)	0.43 (< 0.001)				
Agreement	63.6%				
**Sit-and-reach test (cm)**					
High	90.5%	9.5%		0.0%	
Medium	2.6%	87.2%		10.3%	
Low	0.0%	20.0%		80.0%	
Kappa statistics (*p*-value)	0.78 (< 0.001)				
Agreement	85.9%				
**Squats (reps in 60 s)**					
High	61.5%	38.5%		0.0%	
Medium	10.8%	70.3%		18.9%	
Low	11.8%	11.8%		76.5%	
Kappa statistics (*p*-value)	0.51 (< 0.001)				
Agreement	69.4%				
**The 800 m run test (min)**					
High	57.9%	36.8%		5.3%	
Medium	19.5%	63.4%		17.1%	
Low	0.0%	20.0%		80.0%	
Kappa statistics (*p*-value)	0.47 (< 0.001)				
Agreement	67.1%				
**The 400 m run test (min)**					
High	77.8%		22.2%		0.0%
Medium	12.5%		80.0%		7.5%
Low	0.0%		18.2%		81.8%
Kappa statistics (*p*-value)	0.68 (< 0.001)				
Agreement	79.9%				

*p* < 0.05

## Discussion

The main purpose of the study was to analyze the extent of tracking and the maintenance of tertile classification of several PF tests in adolescent girls measured at two time-points (15 y and 17 y). The main findings of the study are: 1) the largest declines during the follow-up are observed for aerobic capacity, flexibility and speed endurance; 2) the strongest tracking coefficients are obtained for explosive power of lower extremities, flexibility, body composition and speed endurance, and 3) the highest percentage agreements are shown for explosive power of lower extremities, flexibility, speed endurance and body composition.

The tracking coefficients derived from our data our similar, compared to previous studies conducted in youth [13, 18, 22, 23]. A recent longitudinal study showed that the tracking coefficients for similar fitness test assessments ranged between 0.59 and 0.83 between ages 15 y and 18 y [13]. Pate et al. [18] tracked cardiorespiratory and muscular fitness assessed by the Physical Work Capacity 170 test and handgrip strength and found moderate tracking coefficients (*r* = 0.53) in girls. In a study by Matton et al*.* [23], interagecorrelations between adolescence and adulthood were moderate for body composition (*r* = 0.53), muscular endurance of the trunk (*r* = 0.34), explosive power of lower extremities (*r* = 0.59) and speed (*r* = 0.56), while flexibility exhibited moderately high tracking (*r* = 0.76). Additionally, in a group of girls tracked between 6 and 11 y, tracking coefficients were moderate for the standing broad jump (*r* = 0.40), the endurance shuttle run (*r* = 0.42) and sprint (*r* = 0.50) [22]. The averaged tracking coefficient of all seven fitness tests in our study was 0.79, which is similar, compared to one previous study with the same age range (15 y to 18 y; *r* = 0.71) [13]. The adolescent period in our study has been confirmed as a critical time for girls to maintain their PF levels, since they have a longer time span to achieve fitness, compared to boys [13].

As confirmed by previous literature, it is difficult to compare tracking coefficients across studies, due to different methodology, which included tests used to assess the level of PF, sample size, age at first observation, and time points between repeated measures [23, 35]. Nevertheless, previous longitudinal studies have documented that PF components track moderately to moderately high during childhood and adolescence [13, 18, 22, 23]. The nature of higher tracking of PF may be explained by a few mechanisms. First, PF has often been associated with more stable factors that do not change rapidly over time (genotype, morphology) [36]. Second, PF is less sensitive to age-as do opportunities- especially with children in the household [11]. Third, PF is often assessed by objective methods in the literature, reducing the level of measurement error [37].

The second purpose of the study was to analyze the maintenance in a certain tertile (high, medium and low). We found a general trend, indicating that individuals categorized in the lowest tertile at the age of 15 y remained in the lowest tertile at the age of 17 y. The similar stabilization was confirmed for individuals who started in the highest and middle tertile. Previous evidence supports our findings, pointing out that PF track well from childhood to adolescence [13, 16]. However, the percentage agreement in each fitness test in our study is somewhat higher, compared to previous results [13]. Specifically, a study by True et al. [13] showed moderate agreement between the time points, which may be explained by different methodology and the number of time point measurements. Previous studies have highlighted the importance of achieving even a ‘medium’ fitness level, which gives an individual the opportunity to maintain the level of PF in the same category or to move up in a ‘highly fit’ category during pubertal stage [13]. On the other hand, it is relatively unlikely that an individual in the ‘low’ tertile improves to the ‘high’ tertile. Therefore, special interventions and policies aiming to improve the level of PF in the ‘low’ group and at least maintain or enhance the level of PF in the ‘medium’ group should be implemented within the school settings [38].

Adolescence is often highlighted as a period for health-related interventions targeting PF levels. Previous evidence suggests that aerobic- and strength-related activities for improving motor-skill performance and enhance overall PF levels need to be incorporated within an active play, exercise and training [39, 40]. Also, PF should be monitored annually during physical education classes for two purposes: 1) in order to detect a ‘risky’ group of adolescent girls with ‘low’ performance, and 2) to prevent those categorized in the ‘medium’ tertile to drop into the lower tertile. Despite the effort to track PF over time, future research needs to focus on other genetic and environmental factors, which may influence the level and persistence of being in a certain PF category.

This study is not without limitations. Compared to some previous studies, the follow-up period of three years was relatively short and undertaking measures at only two time-points and conclusions should be interpreted with caution. Also, we conducted the study among adolescent girls, and by including the boys, the findings would have been comparable between sexes. Moreover, we did not acknowledge daily or weekly attendance in the movement program. It is possible that an individual was under moderate- or vigorous- intensity physical activity, when the study had been conducted, so this variable could not be accounted for. Lastly, we did not assess maturational level, which might have affected on the development of fitness over time.

## Conclusions

In conclusion, our findings show that PF tracks moderately- to highly-well over a follow-up period of 3 year. The highest tracking coefficients are observed for explosive power of lower extremities, flexibility, body composition and speed endurance. Similarly, the largest percentage agreements are shown for explosive power of lower extremities, flexibility, speed endurance and body composition. Girls maintain their tertile classification of high, moderate and low for each PF test. Thus, the period of adolescence should be a time-point for intervention aiming to enhance or even maintain the level of physical fitness for future acute and long-term health-related benefits.

## Data Availability

The datasets generated and/or analysed during the current study are not publicly available due to the reason that they belong to the Secondary school ‘Gospodarska škola Varaždin’ but are available from the corresponding author on reasonable request.
